# hSulf-1 Gene Exhibits Anticancer Efficacy through Negatively Regulating VEGFR-2 Signaling in Human Cancers

**DOI:** 10.1371/journal.pone.0023274

**Published:** 2011-08-10

**Authors:** Weidan Ji, Jiahe Yang, Duanming Wang, Lu Cao, Weifeng Tan, Haihua Qian, Bin Sun, Qijun Qian, Zhengfeng Yin, Mengchao Wu, Changqing Su

**Affiliations:** 1 Department of Molecular Oncology, Eastern Hepatobiliary Surgical Hospital & Institute, The Second Military Medical University, Shanghai, China; 2 Laboratory of Medical Genetics, Medical College of Soochow University, Suzhou, China; 3 College of Animal Science and Technology, Shihezi University, Xinjiang, China; University of Nebraska Medical Center, United States of America

## Abstract

**Background:**

Human sulfatase 1 (hSulf-1) is a heparin-degrading endosulfatase that desulfates cell surface heparan sulfate proteoglycans (HSPGs) in extracellular matrix and negatively modulates heparin-binding growth factor and cytokine signaling in cell proliferation. But hSulf-1 function is more complicated, and its molecular mechanism has not been well known.

**Principal Findings:**

To further investigate the functions of hSulf-1 gene in regulating the vascular endothelial growth factor receptor (VEGFR) signaling, a series of vectors expressing hSulf-1, hSulf-1 small hairpin RNA (shRNA) and VEGFR-2 shRNA were generated. hSulf-1 re-expression could downregualte the VEGFR-2 phosphorylation and inhibit cancer cell proliferation both in ovarian and hepatocellular cancer cell lines. Knockdown of hSulf-1 expression by hSulf-1 shRNA enhanced the recovery of high levels of phosphorylated VEGFR-2, and knockdown of VEGFR-2 expression by VEGFR-2 shRNA inhibited the proliferation activity of cancer cells *in vitro* to some extent. In human cancer xenografts in nude mice, tumor growth was inhibited markedly after injections of adenovirus expressing hSulf-1, with the tumor inhibition rates of 46.19% and 49.56% in ovarian and hepatocellular tumor models, respectively. hSulf-1 expression significantly reduced tumor microvessel density.

**Conclusions:**

The results demonstrated that hSulf-1 re-expression both in ovarian and hepatocellular cancer cells induces antitumor efficacy by attenuating the phosphorylation of VEGFR-2 and suppressing angiogenesis. Therefore, hSulf-1-mediated antiproliferation and antiangiogenesis could be a reasonable approach for cancer therapy.

## Introduction

Heparan sulfate proteoglycans (HSPGs) in extracellular matrix are important constituents for regulating the heparin-binding growth factor signaling, such as fibroblast growth factor (FGF), epidermal growth factor (EGF) and hepatocyte growth factor (HGF) [1], [2]. The sulfation of *N*-acetylglucosamine residues of HSPGs is critical for the interactions between these factor ligands and their receptor tyrosine kinases at cell surface [3]. Human sulfatase 1 (hSulf-1) was characterized to be a heparin-degrading endosulfatase that functions to desulfate cell surface HSPGs and negatively modulate growth factor and cytokine signaling [4]. hSulf-1 protein is widely expressed in normal tissue, but inactivated in majority of various human cancers, e.g., the ovarian, breast, pancreatic, renal, hepatic, head and neck squamous cell carcinomas [5]–[7]. The loss of heterozygosity, methylation of DNA CpG islands and histone modifications possibly are the main reasons for hSulf-1 inactivation in human cancers [8], [9]. The variant hepatic nuclear factor 1 (vHNF1), encoded by transcription factor 2 gene (TCF2, HNF1beta), was also reported to negatively regulate hSulf-1 expression in ovarian cancer [10]. Re-expression of hSulf-1 in cancer cells effectively results in a decrease of cell proliferation as well as an increase of sensitivity to chemotherapy-induced apoptosis [11]. Therefore, the reported data suggested that hSulf-1 normally functions as a negative regulator in cell proliferation, it may play an important role in cancer therapy.

To investigate the regulatory role of hSulf-1 in heparin-binding growth factor signaling in human cancers, the previous studies identified that hSulf-1 expression can diminish the cascade phosphorylation of a series of kinases including epidermal growth factor receptor (EGFR), extracellular signal-regulated kinase (ERK), mitogen-activated protein kinase kinase (MEK), serine/threonine kinase (AKT) after treatment with exogenously added growth factors, and followed by inactivation of downstream signaling pathways [6], [11], [12]. hSulf-1 is also involved in the inhibition of autocrine-mediated phosphorylation of EGFR-ERK in breast cancer cells induced by serum starvation, and the inhibition of autocrine EGFR-ERK signaling by hSulf-1 results in a reduced expression of Cyclin D1, a decreased S phase fraction and an increased G2-M fraction, and finally leading to the inhibition of cell survival in breast cancer cells [7]. Therefore, loss of hSulf-1 in cancers and cancer cell lines is associated with upregulation of growth factor signaling by enhanced kinase phosphorylation, and the phosphorylation and activation of receptor tyrosine kinases have been implicated in promoting carcinogenesis and development of cancers.

Moreover, the vascular endothelial growth factor (VEGF) and VEGF receptor (VEGFR) are involved in hSulf-1-mediated suppression of cancer cells [6]. We therefore suppose that hSulf-1 may present anticancer potency by inhibiting angiogenesis in most human cancers. The VEGFR family contains three members, VEGFR-1 (Flt-1), VEGFR-2 (KDR/Flk-1) and VEGFR-3 (Flt-4), which are transmembrane tyrosine kinase receptors that regulate the formation of blood and lymphatic vessels. Among these three receptors, VEGFR-2 is generally recognized to have a principal role in mediating VEGF-induced response that directly regulates tumor angiogenesis [13]. In this study, by constructing various vectors carrying the hSulf-1 gene, hSulf-1 small hairpin RNA (shRNA) or VEGFR-2 shRNA, we provided evidence to demonstrate that the hSulf-1 re-expression exhibited a negative effect on cell growth by downregulating VEGFR-2 signaling both in ovarian cancer and hepatocellular carcinoma cell lines. The antitumor efficacy of hSulf-1 was also validated in ovarian and hepatic cancer xenografts in nude mice.

## Results

### Inactivation of hSulf-1 is a common molecular event in majority of human cancers and involves in VEGFR-2 signaling

**Figure 1:**
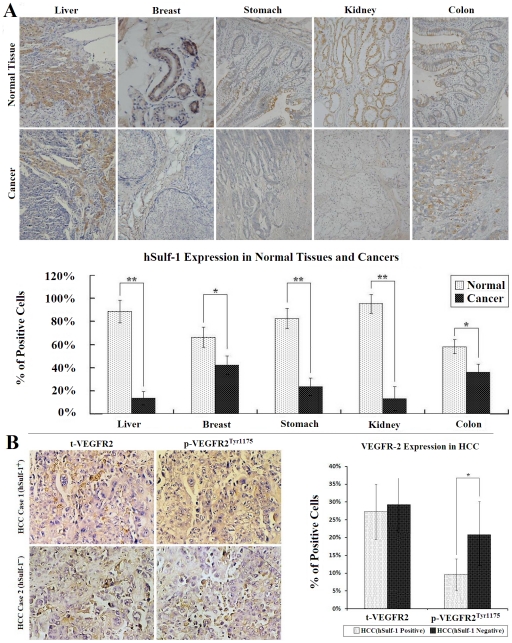
Immunohistochemical examination of hSulf-1 and VEGFR-2 expression in human cancers and normal tissues. (**A**) The specimens, including various cancers and their adjacent normal tissues, were fixed in 10% neutral formaldehyde for 6 h, paraffin-embedded, and sliced into 5 µm-thick sections for hSulf-1 immunohistochemistry. The hepatocellular carcinoma (HCC) cells were negative for hSulf-1 expression, which were surrounded by hSulf-1-positive liver cells. The breast cancer, gastric cancer and renal clear cell carcinoma cells were all negative, and colon cancer cells were positive for hSulf-1 expression; original magnification ×200 (upper panel). The hSulf-1-positive cell percentages in all specimens were counted within 5 high-power fields (original magnification ×400) under microscope, and showed in histograms (lower panel); * P<0.05; ** P<0.01. (**B**) Expression of VEGFR-2, including t-VEGFR2 and p-VEGFR2, in 26 cases of HCC was detected by immunohistochemistry; original magnification ×400 (left panel). The positive cell percentages in 6 hSulf-1-positive and 20 hSulf-1-negative HCC were counted within 5 high-power fields (original magnification ×400) under microscope, and showed in histograms (right panel); * P<0.05.

The hSulf-1 protein is widely expressed in normal tissue and functions to negatively modulate growth factor signaling. To demonstrate its inactivation in majority of various human cancers, we examined hSulf-1 expression in many types of human cancer specimens by immunohistochemistry. In the epithelial cells of normal tissues, hSulf-1 was positive with a positive rate of 100.0%. But in their corresponding cancers, hSulf-1expression was suppressed obviously. The positive rates of hSulf-1 were 23.1% (6/26), 16.7% (2/12), 31.8% (7/22), 11.1% (1/9), 44.4% (8/18) in hepatocellular, breast, gastric, renal and colon cancers, respectively (Fig. 1A).

The evident effect of hSulf-1 is to diminish the cascade phosphorylation of a series of receptor tyrosine kinases, which was demonstrated in VEGF and VEGFR signaling [6]. We therefore explored the expression of total VEGFR-2 (t-VEGFR2) and phosphorylated VEGFR-2 on Tyr1175 (p-VEGFR2^Tyr1175^) in tumor specimens (Fig. 1B). Among 26 cases of hepatocellular carcinoma, there is an obvious decrease of p-VEGFR2^Tyr1175^ level in the hSulf-1-positive hepatocellular carcinoma than that in the hSulf-1-negative hepatocellular carcinoma (P<0.05), but no difference of t-VEGFR2 expression between them (P>0.05).

### Adenovirus-mediated hSulf-1 re-expression downregulates the phosphorylation of VEGFR-2 in cancer cells

**Figure 2:**
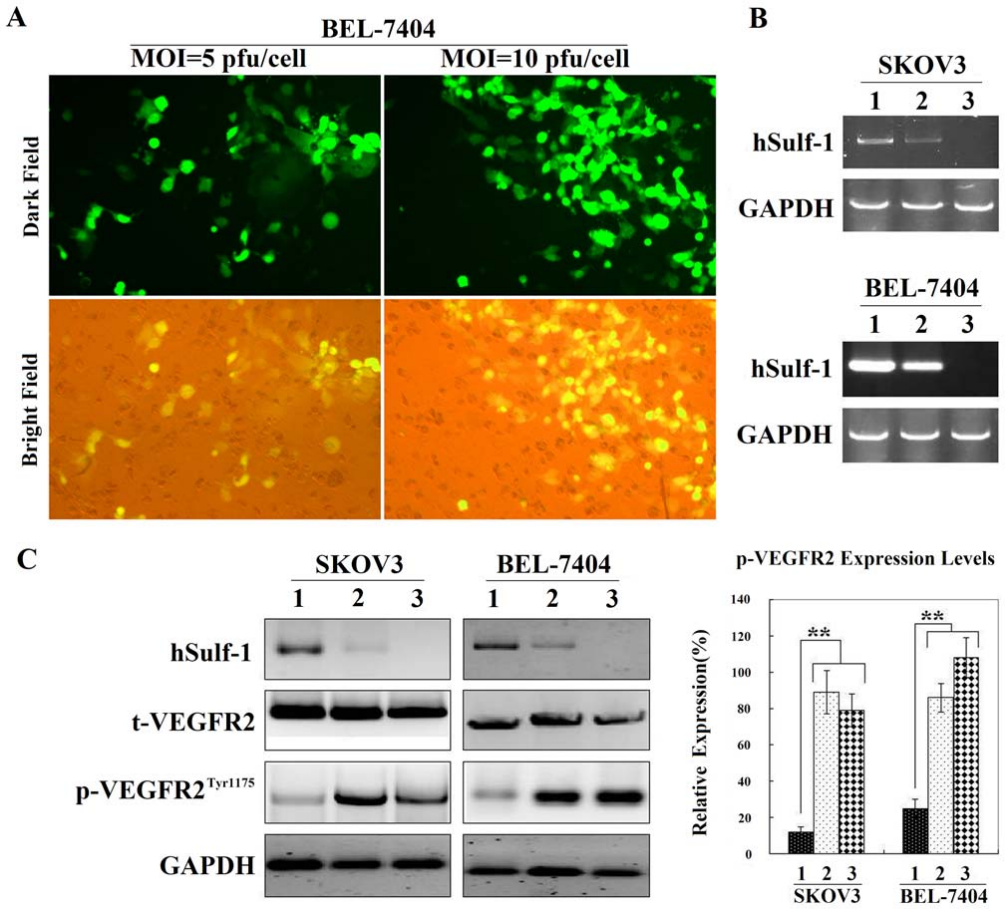
Re-expression of hSulf-1 in cancer cells decreased the p-VEGFR2^Tyr1175^ levels. (A) BEL-7404 cells were infected with the control adenovirus Ad5-EGFP at MOIs of 5 and 10 pfu/cell, and the percentages of EGFP-positive cells were observed under a fluorescent microscope, original magnification ×400. (**B**) RT-PCR was used to identify hSulf-1 expression mediated by adenovirus Ad5-hSulf1. 1, Cells infected with Ad5-hSulf1 at an MOI of 10 pfu/cell; 2, Cells infected with Ad5-hSulf1 at an MOI of 10 pfu/cell and then transfected with hSulf-1 shRNA vector at a concentration of 20 µg/10^5^ cells; 3, Parental cells. (**C**) Expression of hSulf-1, t-VEGFR2 and p-VEGFR2^Tyr1175^ was identified by western blotting. Glyceraldehyde phosphate dehydrogenase (GAPDH) was used as a loading control. Densitometric analysis was performed to show the expression levels of p-VEGFR2^Tyr1175^ in cancer cells, normalized with the GAPDH density. Columns are the mean of three separate analyses; bars = SD; **P<0.01.

To test the infection efficiency of adenovirus, BEL-7404 cancer cells were infected with the control adenovirus Ad5-EGFP carrying a reporter gene of enhanced green fluorescent protein (EGFP) and observed forty-eight h after infection under a fluorescent microscope. The percentages of EGFP-positive cells were 42.67±12.25% and 86.33±26.48% at multiplicities of infection (MOI) of 5 and 10 pfu/cell, respectively (Fig. 2A).

The parental cancer cell lines, SKOV3 and BEL-7404, were negative for hSulf-1 expression. After 48 h post-infection of Ad5-hSulf1 at an MOI of 10 pfu/cell, cancer cells were positive for hSulf-1, and the hSulf-1 shRNA could downregulate the hSulf-1 expression level (Fig. 2B). Since the hSulf-1 gene can diminish the phosphorylation of kinases involved in many growth factor signaling pathways, we examined the expression levels of t-VEGFR2 and p-VEGFR2^Tyr1175^. Compared with the parental cancer cells, the level of t-VEGFR2 remained no change in the Ad5-hSulf1 infected cells. However, the level of p-VEGFR2^Tyr1175^ had an obvious decrease after infection of Ad5-hSulf1. When the hSulf-1 shRNA was transfected into the Ad5-hSulf1 infected cancer cells, hSulf-1 expression was re-inhibited, and the content of p-VEGFR2^Tyr1175^ recovered nearly to the normal levels (Fig. 2C).

### Adenovirus-mediated hSulf-1 reactivation inhibits cancer cell proliferation

**Figure 3:**
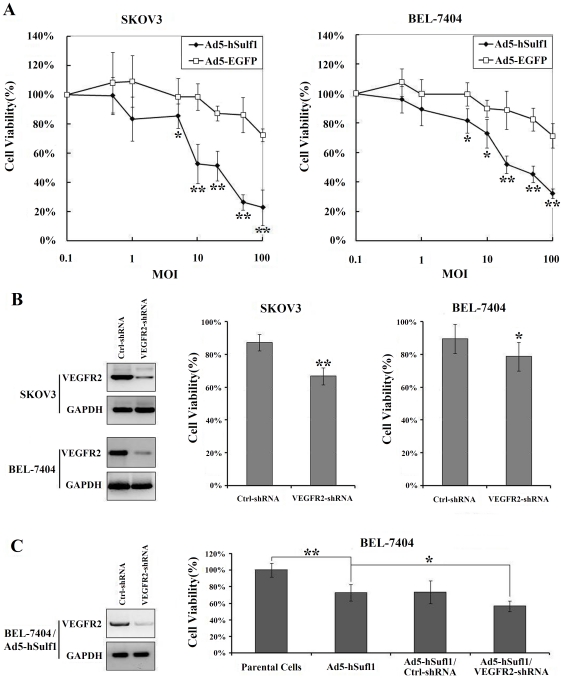
Cell viability was measured by MTT assay. (**A**) SKOV3 and BEL-7404 cancer cells were infected with Ad5-hSulf1 at different MOIs. Ad5-EGFP was used as a control adenovirus. (**B**) SKOV3 and BEL-7404 cancer cells were transfected with VEGFR-2 shRNA vector at concentration of 20 µg/well. Forty-eight h after transfection, VEGFR-2 expression was detected by western blotting and cell viability was detected by MTT assay. The negative control shRNA (Ctrl-shRNA) was used as a negative control; *P<0.05; **P<0.01. (**C**) BEL-7404 cancer cells were infected with Ad5-hSulf1 at MOI of 10 pfu/ml and then transfected with VEGFR-2 shRNA vector at concentration of 20 µg/10^5^ cells, then VEGFR-2 expression and cell viability were detected. BEL-7404 parental cells were used to represent the maximum amount of cell growth to produce the percent viability; *P<0.05; **P<0.01.

Because the loss of hSulf-1 is a common molecular event in majority of human cancers, we reactivated hSulf-1 expression by infection of adenovirus carrying the hSulf-1 gene in different cancer cell lines and examined cell proliferation. Compared with the control adenovirus Ad5-EGFP, Ad5-hSulf1 exerted an obvious inhibition effect on cancer cell proliferation with MOI-dependent manner. When MOI was more than 20 pfu/cell, the cell viability was decreased to lower than 50% in the Ad5-hSulf1 infected cancer cells, whereas, the cell viability was more than 80% in the Ad5-EGFP infected cancer cells (Fig. 3A).

Cancer cells cultured in 96-well plates were transfected with the vectors containing the VEGFR-2 shRNA and negative control shRNA at concentration of 20 µg/well. The expression of VEGFR-2 was examined by Western blotting, and cell viability was measured by 3-(4,5-Dimethylthiazol-2-yl)-2,5-diphenyltetrazolium bromide (MTT) assay. Compared with the negative control shRNA, the VEGFR-2 shRNA inhibited VEGFR-2 expression and decreased cell viability to some extent (Fig. 3B). To demonstrate if VEGFR-2 knockdown under conditions of hSulf-1 overexpression has the same effect on cell viability, BEL-7404 cancer cells, which were infected with Ad5-hSulf1 at an MOI of 10 pfu/cell, were transfected with VEGFR-2 shRNA vector at a concentration of 20 µg/10^5^ cells to knockdown the expression of VEGFR-2 (Fig. S1), the results showed that BEL-7404 cell viability after transfection of VEGFR-2 shRNA was further decreased in the context of hSulf-1 effect (Fig. 3C).

### Adenovirus-mediated hSulf-1 gene therapy exhibits a potent antitumor efficacy by antiangiogenesis in human cancer xenografts in nude mice

**Figure 4:**
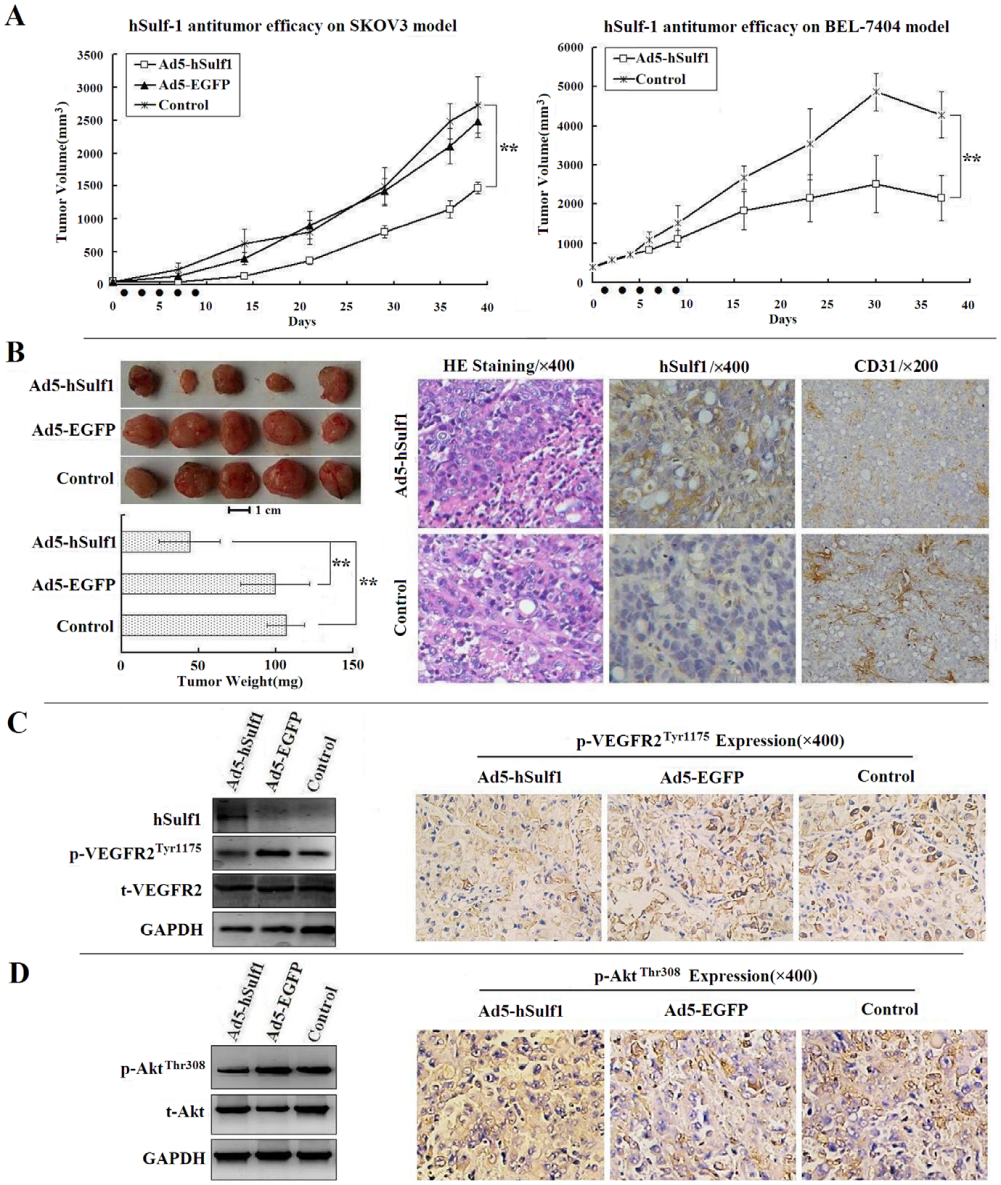
hSulf-1-mediated antitumor efficacy in human cancer xenografts in nude mice. (**A**) SKOV3 and BEL-7404 models, 5 mice per group, suppression effect of Ad5-hSulf1 on tumor growth was analyzed, compared with the control group or the negative adenovirus Ad5-EGFP group; Black spots on X-axis presented the time points of adenovirus injections; **P<0.01. (**B**) Pathological examination of SKOV3 xenograft tumors. Comparison of tumor weight in SKOV3 models (left panel); Bar = 1 cm; **P<0.01 versus the control or Ad5-EGFP groups. By hematoxylin and eosin staining (HE) and immunohistochemical examinations, the positive cell percentages for hSulf-1, the microvessel density (MVD) count labeled by CD31 antibody, were quantified within 5 high-power fields (original magnification ×400) under microscope. After injections of Ad5-hSulf1, tumor cells were positive for hSulf-1 expression in cytoplasm. Accordingly, the count of MVD was decreased markedly, compared with that of in the control group. (**C**, **D**) The total VEGFR-2 and phosphorylated VEGFR-2 (C), and total AKT and phosphorylated AKT (D) were identified by western blotting (left panel) and immunohistochemistry (right panel) in Ad5-hSulf1 treated SKOV3 xenograft tumors, compared with the control and Ad5-EGFP groups.

Reactivation of hSulf-1 function in cancer cells can inactivate the downstream growth factor signaling pathways, therefore, hSulf-1 is a novel target for cancer gene therapy. Hereby, we constructed an adenovirus vector expressing the wild type hSulf-1 gene, Ad5-hSulf1. Its antitumor efficacy was validated both in ovarian and hepatocellular cancer xenografts in nude mice (Fig. 4A). After intratumoral injections of viruses at a total dose of 10^9^ pfu per mouse, suppression of tumor growth in the Ad5-hSulf1 treated group was more effective, with the tumor inhibition rates of 46.19% and 49.56% in SKOV3 and BEL-7404 models, respectively, compared with the blank control group (P<0.01). There was no difference between the negative virus control group and the blank control group (P>0.05).

At the end of observation period, mice bearing SKOV3 xenografts were sacrificed and tumors were removed and weighed. The tumor weights of the Ad5-hSulf1 group were lower than that of the other two groups (Fig. 4B, left panel). The paraffin-embedded tumor sections were examined immunohistochemically. In the blank control group, cancer cells were negative for hSulf-1 expression. But in the Ad5-hSulf1 group, cancer cells re-expressed hSulf-1 protein. A quantitative analysis of microvessel density (MVD) was performed by CD31 immunohistochemistry. The MVDs in tumor tissues were 24.67±6.51 and 52.33±12.34 in the Ad5-hSulf1 and control groups, respectively (Fig. 4B, right panel). There was a significant difference between them (P<0.05).

As the hSulf-1 gene exerts a wide role in regulating multiple pathways by inhibiting the phosphorylation of intracellular tyrosine kinases which could be critical in tumor cell proliferation and tumor angiogenesis, we therefore examined the expression of downstream proteins, including VEGFR-2 and serine/threonine kinase (AKT) in xenograft tumors. The results showed that the expression of p-VEGFR2^Tyr1175^ and phosphorylated AKT on Thr308 (p-AKT^Thr308^) was downregulated in the Ad5-hSulf1 group examined by Western blotting and immunohistochemistry (Fig. 4C, D).

## Discussion

The sulfation of cell surface HSPGs is thought to play an important role in regulating the heparin-binding growth factor signaling in extracellular matrix [14], [15]. The hSulf-1 protein is an arylsulphatase activity enzyme that can negatively regulate the sulfation state of HSPGs [16]. Strong evidence demonstrated that hSulf-1 normally functions to desulfate cell surface HSPGs and downregulate the receptor tyrosine kinase signaling to effectively abrogate cell growth and survival [4], [17]. This process plays a distinct part in the inhibition of malignant transformation and cancer cell growth [18], [19]. Therefore, hSulf-1 is considered as a tumor suppressor gene. Previous studies showed that hSulf-1 is inactivated in majority of human cancers through either genetic mechanisms, such as deletion and mutation, or through epigenetic mechanisms, such as DNA methylation and histone deacetylation [12], [20], [21]. We also demonstrated immunohistochemically that hSulf-1 expression was downregulated in 87 cases of clinical specimens including hepatocellular, breast, gastric, renal and colon cancers, compared with their adjacent normal tissues. Due to the reasons that hSulf-1 has complicated functions, and its molecular mechanism has not been well known yet, in these studies we tested if hSulf-1-mediated inhibition of VEGFR signaling is associated with antiproliferation and antiangiogenesis in cancers.

Both primary lesions and metastatic tumors must develop a new vascular supply in order to support cancer cell expansion and dissemination. Most cancer cells can express both VEGF ligand and VEGFR that act in an autocrine loop to directly stimulate tumor angiogenesis [22]. Angiogenesis is a rate-limiting step in cancer growth, progression and metastasis. VEGF is a critical mediator of angiogenesis, which is well-balancedly expressed in most tissues and cell types, but highly up-regulated in tumors [23]. Binding of VEGF to its receptor results in the receptor autophosphorylation and subsequent activation of a series of tyrosine kinases, then activates multiple downstream proteins that play functional roles in cell survival, cell proliferation, vascular permeability and stabilization of new blood vessels [24]–[26]. Therefore, the phosphorylation-mediated activation of VEGFR is an important process for the regulation of cancer growth. Because hSulf-1 catalyzes the desulfation of HSPGs, therefore it affects the binding ability of heparin-binding factors to their receptors in the EGFR, ERK1/2, MEK, PI3K/AKT signaling pathways, and depresses the phosphorylation and activation of receptor tyrosine kinases. These signaling pathways were all involved in angiogenic process [27]–[29]. Highly sulfated HSPGs potentiate the interaction between VEGF and VEGFR-2, then phosphorylate and activate VEGFR-2. In this process, the expression of VEGF and VEGFR-2 might not be affected by sulfation or desulfation of HSPGs [30]. It was found that the enhancement of VEGFR-2 phosphorylation on Tyr1175 was known to be essential for VEGF-dependent activation of MAPK signaling and angiogenesis [31]. To verify the negative effect of hSulf-1 on cancer angiogenesis, we generated adenovirus expressing hSulf-1. Adenovirus-mediated hSulf-1 expression not only downregualted the levels of phosphorylated VEGFR-2 but also inhibited the proliferation of cancer cells both in ovarian and hepatocellular cancer cell lines. Knockdown of hSulf-1 expression by hSulf-1 shRNA vector enhanced the recovery of high levels of phosphorylated VEGFR-2, indicating that hSulf-1 regulates the phosphorylation and activation of VEGFR-2. However, inhibition of cancer cell proliferation *in vitro* by hSulf-1 re-expression might be mainly due to the desulfation of HSPGs and inactivition of many growth factor signaling pathways. However, when we used the VEGFR-2 shRNA to silence the expression of VEGFR-2 in ovarian and hepatocellular cancer cells, the cell viability was decreased to some extent, demonstrating that the VEGFR-2 signaling participates in the regulation of cancer cell proliferation, and the antiproliferation effect of hSulf-1 on cancer cells is partly due to the inhibition of VEGFR-2 signaling. When BEL-7404 cancer cells were infected with Ad5-hSulf1 to re-express hSulf-1 and then transfected with VEGFR-2 shRNA to silence VEGFR-2 expression, the cell viability was further decreased, exactly demonstrating that there is another mechanism involved in VEGFR-2 activation and cancer cell proliferation in the context of hSulf-1 effect.

To evaluate the effect of hSulf-1 on tumor growth, we treated human cancer xenografts in nude mice with adenovirus expressing hSulf-1. The results found that the tumor growth was inhibited after treatment. The tumor inhibition rates were 46.19% and 49.56% in ovarian and hepatocellular tumor models, respectively. Re-expression of hSulf-1 resulted in downregulation of phosphorylated VEGFR-2 and phosphorylated AKT, then significantly reduced tumor microvessel density, indicating that hSulf-1 expression was associated with antiangiogenesis.

Conclusively, hSulf-1 is a sulphatase that functions to desulfate cell surface HSPGs. It can inhibit the downstream kinase phosphorylation with a broad spectrum and negatively regulate the receptor tyrosine kinase signaling. This study gave a convincing evidence to demonstrate that hSulf-1 re-expression both in ovarian and hepatocellular cancer cells attenuates the phosphorylation of VEGFR-2, then suppresses cancer cell proliferation and angiogenesis, finally induces antitumor efficacy. Therefore, our data suggested that hSulf-1-mediated antiproliferation and antiangiogenesis could be a reasonable approach for cancer therapy.

## Materials and Methods

### Examinations of hSulf-1 and VEGFR-2 expression in clinical cancer specimens

Expression of hSulf-1 was detected by immunohistochemistry in 87 cases of clinical cancer specimens, including 26 hepatocellular carcinomas, 12 breast cancers, 22 gastric cancers, 9 renal cancers, 18 colon cancers, and their adjacent normal tissues. VEGFR-2, including t-VEGFR2 and p-VEGFR2^Tyr1175^, was also detected in 26 hepatocellular carcinomas by immunohistochemistry. The specimens were fixed in 10% neutral formaldehyde for 6 h, paraffin-embedded, and sliced into 5 µm-thick sections for immunohistochemistry with a rabbit anti-hSulf-1 antibody (Abcam inc., Cambridge, MA), a rabbit anti-VEGFR-2 antibody and a rabbit anti-Phospho-VEGFR2^Tyr1175^ antibody (Cell Signaling Technology, Inc., Danvers, MA). Approval for the use of clinical specimens was given by the Research Ethics Committee, The Second Military Medical University, and we have obtained the written informed consent from all participants involved in the study.

### Preparation of vectors and adenoviruses

The plasmid pcDNA3.1-hSulf1 containing the whole-length hSulf-1 cDNA and the vector pSUPER.retro.puro containing the hSulf-1 shRNA (19 oligonucleotide pairs targeting hSulf-1 cDNA positions 294–312: GTATGTGCACAATCACAAT) were kindly gifted from Viji Shridhar (Department of Experimental Pathology, Mayo Clinic Cancer Center, Rochester, MN). The vector pGenesil-1.1 containing the VEGFR-2 shRNA (19 oligonucleotide pairs targeting VEGFR-2 cDNA positions 525–543: CAGAATTTCCTGGGACAGC) or the negative control shRNA (19 oligonucleotide pairs: gacttcataaggcgcatgc) were purchased from Wuhan Genesil Biotechnology Co., Ltd. (Wuhan, China).

To recombine adenovirus vector, pcDNA3.1-hSulf1 was used to amplify the hSulf-1 cDNA with the primers P1 (5′-cgggatccaccatgaagtattcttgc-3′) and P2 (5′-gcgtcgacttaaccttcccatccatcccataac-3′). The PCR product was digested with BamHI and SalI, then inserted into the adenovirus plasmid pDC315 (Microbix Biosystems, Ontario, Canada) to generate pDC315-hSulf1. The plasmids pDC315-hSulf1 and pDC315-EGFP were transfected, respectively, into HEK293 cells (Microbix Biosystems, Ontario, Canada) using the PolyFect Transfection Reagent (QIAGEN Inc., Valencia, CA) together with the adenovirus packaging plasmid pBHGloxdelE13cre (Microbix Biosystems, Ontario, Canada). After a homologous recombination in HEK293 cells, we obtained adenoviruses named Ad5-hSulf1 and Ad5-EGFP. Ad5-EGFP was used as the virus control. All adenoviruses were amplified in HEK293 cells and purified by ultra-centrifugation on cesium chloride (CsCl) gradients. The viral titers were detected with the Tissue Culture Infectious Dose 50 (TCID50) method [32] established by Qbigene Inc. (IIIkich, France), and showed as plague-forming units per milliliter (pfu/ml).

### Cell culture and transfectants

The ovarian cancer cell line SKOV3 (American Type Culture Collection, Manassas, VA), the hepatocellular carcinoma cell line BEL-7404 (Institute of Cell Biology, Chinese Academy of Sciences, Shanghai, China) were cultured in media according to the providers' recommendations. When cells were in logarithmic phase, they were infected with adenoviruses (Ad5-hSulf1 or Ad5-EGFP) at MOIs of 0.5, 1, 5, 10, 20, 50, 100 pfu/cell, and harvested 48 h after infection. The virus-infected cells and their parental cells were transfected with hSulf-1 shRNA and VEGFR-2 shRNA vectors using the PolyFect Transfection Reagent (QIAGEN Inc., Valencia, CA) according to the provider's protocol. Twenty-four h later, puromycin (3 pg/ml) or G418 (400 µg/ml) was added to select hSulf-1 shRNA transfectants or VEGFR-2 shRNA transfectants, respectively. After continuously cultured for 24 h, cells were harvested and the silence of the target gene expression was tested.

### *In vitro* examination of correlative factor expression

Cancer cells, including the parental, virus-infected and shRNA transfected cells, were harvested 48 h after infection or transfection. Total RNA was extracted from 10^5^ cells with TriZol reagent (Invitrogen, Carlsbad, CA) and used to amplify hSulf-1 expression by reverse transcription polymerase chain reaction (RT-PCR), with the primers P3 (5′- ccaccttcatcaatgcctt-3′) and P4 (5′- ccttgaccagtccaaactgc-3′). The amplified fragments were 762 bp. Glyceraldehyde phosphate dehydrogenase (GAPDH) was amplified with the primers P5 (5′-accacagtccatgccatcac-3′) and P6 (5′-tccaccaccctgttgcttgta-3′) as an inner control. Total protein was extracted from 10^5^ cells by M-PER Mammalian Protein Extraction Reagent (PIERCE, Rockford, IL) and investigated by western blotting as previously described [33], with the indicated primary antibodies, including the rabbit anti-VEGFR-2 and rabbit anti-Phospho-VEGFR-2^Tyr1175^ (Cell Signaling Technology, Inc., Danvers, MA).

### Cell viability by MTT assay

The parental, virus-infected and shRNA transfected cells were diluted at concentration of 10^5^ cells/ml, and plated at density of 100 µl/well in 96-well plates. Cell viability was measured by MTT assay using Cell Proliferation Kit I (Roche Molecular Biochemicals, Indianapolis, IN) as described above [34]. Average absorbance for each sample was examined with a microplate reader (Model 550, BIO-RAD Laboratories, Tokyo, Japan) at a wavelength of 570 nm with a reference wavelength of 655 nm.

### Animal models and *in vivo* experiments

SKOV3 and BEL-7404 cells were subcutaneously injected into the right flanks of BALB/c (nu/nu) mice (Shanghai Experimental Animal Center, Chinese Academy of Sciences, Shanghai, China), 10^7^ cells per mouse, to establish xenografts. Three weeks later, mice were separated randomly into 3 groups: the Ad5-hSulf1, Ad5-EGFP and control groups, 5 mice per group. Mice in the Ad5-hSulf1 and Ad5-EGFP groups were given 5 intratumoral injections, one injection every other day, with a total dose of 10^9^ pfu viruses per mouse. Mice in the control group were given the same volume of viral preservation solution (10 mmol/L Tris-HCl pH 8.0, 2 mmol/L MgCl_2_, 4% sucrose). Tumor size was measured regularly, and tumor volume was estimated with the formula “*a*×*b* ^2^×0.5”, in which *a* and *b* represent the maximal and minimal diameters. Mice were euthanized at the end of observation period, and tumors were removed, weighed and fixed in 10% neutral formaldehyde for 6 h. The paraffin-embedded consecutive sections were cut for examining the expression of hSulf-1, t-VEGFR2 p-VEGFR2^Tyr1175^ and t-AKT, p-AKT^Thr308^ by immunohistochemistry and western blotting. The rabbit anti-Phospho-AKT^Thr308^ was purchased from Santa Cruz Biotechnology, Inc. (Santa Cruz, CA). The MVD value in tumor tissues was performed by CD31 immunohistochemistry using a rat anti-mouse CD31 monoclonal antibody (BD Biosciences Pharmingen, San Diego, CA). The positive cell percentages and MVD value in tumors were counted within 5 random high-power fields (original magnification ×400) under microscope, and shown as mean ± standard deviation (SD) [35]. The animal welfare guidelines for the care and use of laboratory animals were followed and the experimental protocol was approved by the Animal Care Committee, The Second Military Medical University, and the approval ID for this study is SCXK2009-0003.

### Statistical analysis

The experimental data from 3 times of independent *in vitro* experiments, as well as *in vivo* experimental data from 5 mice per group, were analyzed statistically by the student *t*-test at a significance level of P<0.05.

## Supporting Information

Figure S1 **Transfection efficiency of VEGFR-2 shRNA with pGenesil-1.1 vector containing a reporter gene of enhanced green fluorescent protein (EGFP).** Cancer cells were transfected with VEGFR-2 shRNA vector at concentration of 20 µg/10^5^ cells, forty-four h later after transfection, the percentages of EGFP-positive cells were 26.33±8.22% and 38.67±16.15% in SKOV3 and BEL-7404 cells, respectively, when counted under a fluorescent microscope, original magnification ×200.
(TIF)
