# Supplementary figures and images for: hSulf-1 Gene Exhibits Anticancer Efficacy through Negatively Regulating VEGFR-2 Signaling in Human Cancers
Source: PLoS One. 2011 Aug 10;6(8):e23274. doi: 10.1371/journal.pone.0023274 (PMC3154391; doi:10.1371/journal.pone.0023274)

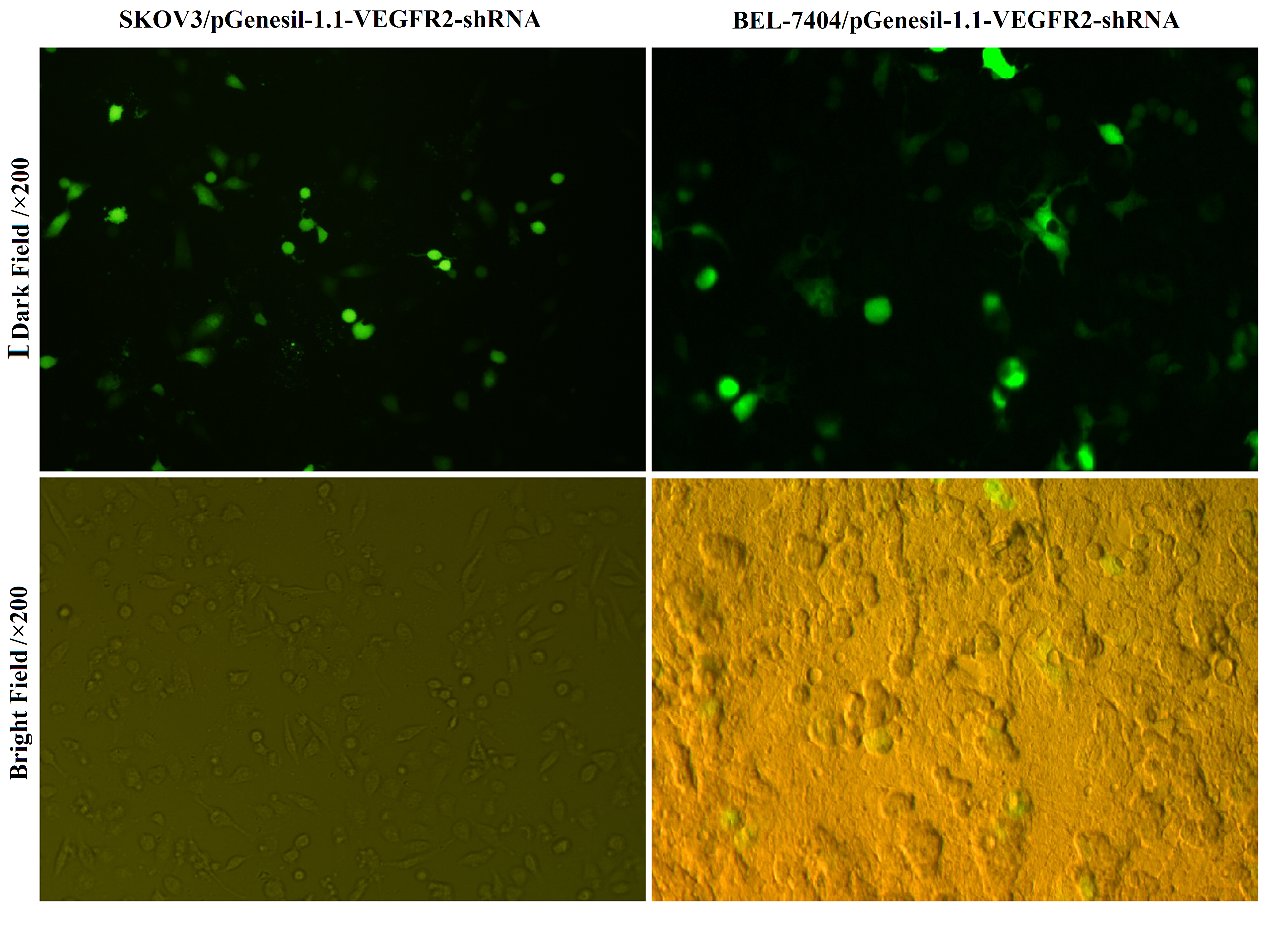

Supplement: Figure S1 — Transfection efficiency of VEGFR-2 shRNA with pGenesil-1.1 vector containing a reporter gene of enhanced green fluorescent protein (EGFP). Cancer cells were transfected with VEGFR-2 shRNA vector at concentration of 20 µg/105 cells, forty-four h later after transfection, the percentages of EGFP-positive cells were 26.33±8.22% and 38.67±16.15% in SKOV3 and BEL-7404 cells, respectively, when counted under a fluorescent microscope, original magnification ×200. (TIF) [file pone.0023274.s001.tif]
